# Water vapor sorption and glass transition temperatures of phase-separated amorphous blends of hydrophobically-modified starch and sucrose

**DOI:** 10.1016/j.dib.2018.08.105

**Published:** 2018-09-11

**Authors:** Job Ubbink, Thomas Zwick, David Hughes, Gabriela Badolato Bönisch

**Affiliations:** a Food Science and Nutrition Department, California Polytechnic State University, 1 Grand Ave., San Luis Obispo, CA 93407, USA; b DSM Nutritional Products Ltd, Research Center Formulation & Application, P.O. Box 2676, 4002 Basel, Switzerland; c H. H. Wills Physics Laboratory, University of Bristol, Tyndall Avenue, Bristol BS8 1TL, United Kingdom

## Abstract

This article contains water vapor sorption data obtained on amorphous blends of octenyl succinic acid-modified (denoted as hydrophobically modified starch; HMS) and sucrose (S) in the anhydrous weight HMS/S ratios between 100/0 and 27/75. The water vapor sorption data was obtained gravimetrically. The amorphous state of the blends was confirmed by X-ray diffraction. The glass transition temperatures of the phase-separated blends are listed; the blends show phase separation into a sucrose-rich phase and a HMS-rich phase, the composition of which varies with the blend ratios. The sucrose-rich phase is characterized by a glass transition temperature *T*_g,lower_ that is 40 to 90 K lower than the glass transition temperature *T*_g,upper_ of the HMS-rich phase.

**Specifications table**

**Table:** 

Subject area	*Physical chemistry*
More specific subject area	*Hydrocolloids, carbohydrate polymers, phase transitions*
Type of data	*Table (Water vapor sorption, glass transition data), figure (X-ray diffraction, Water vapor sorption isotherms*
How data was acquired	*Water vapor sorption data (gravimetric analysis); X-ray diffraction data (Phillips X’pert Pro diffractometer (Panalytical)); Differential Scanning Calorimetry (Discovery DSC, TA Instruments)*
Data format	*Analyzed data*
Experimental factors	*Spray-dried blends of Octenyl succinic acid-modified starch and sucrose in the anhydrous weight ratios 100/0, 90/10, 80/20, 60/40, 45/55 and 25/75.*
Experimental features	*Spray-dried blends were water activity-equilibrated at water activities 0.11, 0.22, 0.33, 0.43, 0.54, 0.68 and 0.75 (T = 298 K). Water vapor sorption was determined gravimetrically until equilibrium was achieved (1200 hours). Water activity-equilibrated samples were analyzed for eventual crystallinity by X-ray diffraction and for the glass transitions of the phase separated blends (sucrose-rich and modified starch-rich phases) by Differential Scanning Calorimetry.*
Data source location	*NA*
Data accessibility	*NA*
Related research article	*D. J. Hughes, G. Badolato Bönisch, T. Zwick, C. Schäfer, C. Tedeschi, B. Leuenberger, F. Martini, G. Mencarini, M. Geppi, M. A. Alam, J. Ubbink, Phase separation in amorphous hydrophobically-modified starch - sucrose blends: Glass transition, matrix dynamics and phase behavior, Carbohydrate Polymers (in press)*

**Value of the data**

• We present a broad set of water vapor data on blends of hydrophobically modified starch and sucrose with a systematic variation in composition. The water vapor data are obtained in the range between 0.11 and 0.75 at *T* = 298 K.
• Data on the glass transition temperatures of the phase-separated blends is valuable in the context of the understanding of the phase behavior of amorphous phase-separated systems.
• These data allow the exploration of the effect of composition on water vapor sorption behavior in the glass transition range.

## 1 Data

**Fig. 1:**
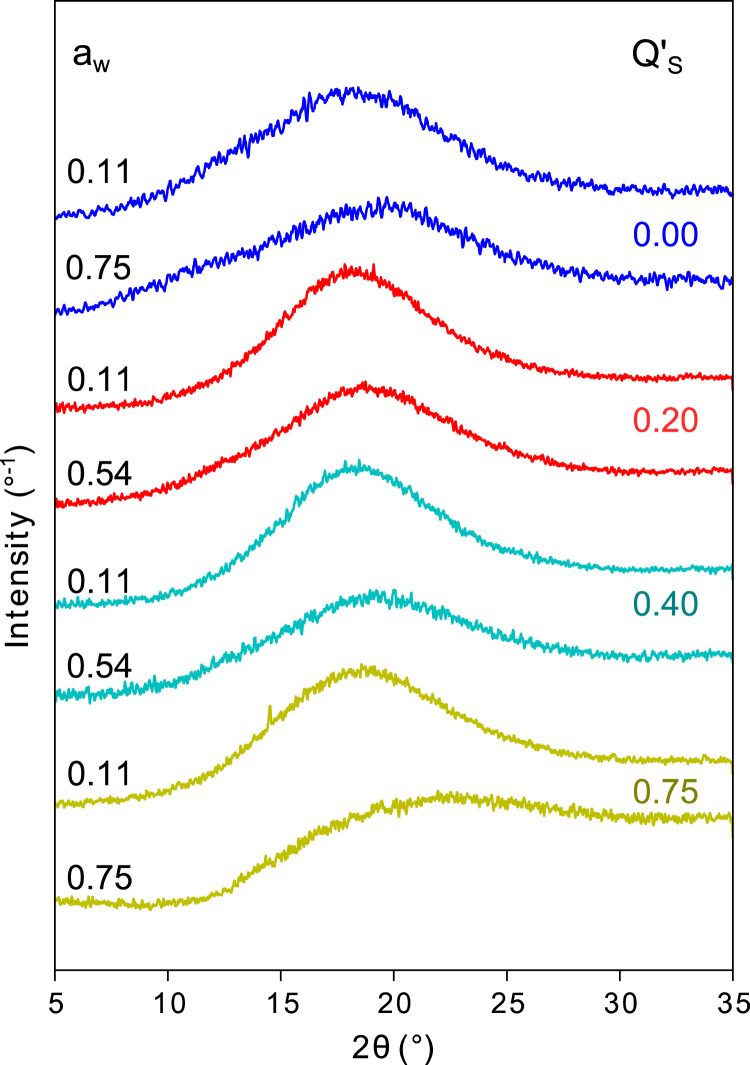
Normalized powder X-ray diffraction profiles of spray-dried HMS/S blends equilibrated at selected water activities. Q׳s is the weight fraction of sucrose in the HMS/S blends on anhydrous basis.

Spray-dried blends of hydrophobically-modified starch and sucrose were water activity-equilibrated at water activities 0.11, 0.22, 0.33, 0.43, 0.54, 0.68 and 0.75 (*T* = 298 K). Water vapor sorption was determined gravimetrically until equilibrium was achieved (1200 h); the data is reported in Table 1. Water activity-equilibrated samples were analyzed for eventual crystallinity by X-ray diffraction (Fig. 1) and for the glass transitions of the phase separated blends (sucrose-rich and modified starch-rich phases) by Differential Scanning Calorimetry (Tables 3 and 4).

**Table 1:** Equilibrium water content of water activity equilibrated HMS-S blends at *T* = 298 K.

***a***_**w**_**(*T* = 298 K)**	**Water content on wet basis [wt.%]**					
	**100/0**	**90/10**	**80/20**	**60/40**	**45/55**	**25/75**
0.11	6.1	4.7	3.3	2.4	2.3	2.3
0.22	7.7	6.0	4.6	3.9	4.4	4.6
0.33	9.1	7.1	5.7	5.5	6.4	6.8
0.43	10.4	8.3	7.4	7.9	9.2	10.1
0.54	11.7	9.7	9.2	10.5	12.0	13.6
0.75	15.9	15.1	16.9	19.6	22.9	29.8

**Table 2:** GAB fitting coefficients for the water vapor sorption isotherms of the HMS-S blends.

**GAB coefficient**	**HMS-S blend**					
	**100/0**	**90/10**	**80/20**	**60/40**	**45/55**	**25/75**
*K*	0.767	0.91	1.03	0.99	1.00	1.06
*C*	27.2	33.5	12.8	3.06	2.58	2.50
*W*_m_	8.25	5.67	4.77	6.99	8.45	7.25

**Table 3:** Water content and parameters associated with the glass transition fitting, as described in Section 2.4 of [2], for water activity equilibrated HMS-S blends. Q׳s is the weight fraction of sucrose in the HMS-S blends (on anhydrous basis), Q_w_ is the weight fraction of water in the matrices, ∆*C*_p,lower_ and ∆*C*_p,upper_ are the changes in heat capacity associated with the lower and upper glass transitions, *T*_g,lower_ and *T*_g,upper_ are the glass transition temperatures and of the sucrose-rich and the HMS-rich phases, respectively, and ∆*T*_g,lower_ and ∆*T*_g,lower_ are the widths of the two glass transitions.

***Q***_**s**_**[dimensionless]**	***a***_**w**_**[dimensionless]**	***Q***_**w**_**[dimensionless]**	**∆*C***_**p,lower**_**[J g**^**−1**^**K**^**−1**^**]**	***T***_**g,lower**_**[K]**	**∆*T***_**g,lower**_**[K]**	**∆*C***_**p,upper**_**[J g**^**−1**^**K**^**−1**^**]**	***T***_**g,upper**_**[K]**	**∆*T***_**g,upper**_**[K]**
0	0.11	6	–	–	–	0.16 ± 0.01	405 ± 1	13 ± 1
	0.22	7.7	–	–	–	0.16 ± 0.01	388 ± 1	16 ± 1
	0.33	9.1	–	–	–	0.17 ± 0.01	377 ± 1	15 ± 1
	0.43	10.4	–	–	–	0.17 ± 0.01	366 ± 1	16 ± 1
	0.54	11.7	–	–	–	0.17 ± 0.01	357 ± 1	14 ± 1
	0.68	14.1	–	–	–	0.19 ± 0.01	337 ± 1	17 ± 1
	0.75	15.9	–	–	–	0.19 ± 0.01	325 ± 1	18 ± 1
0.1	0.11	4.7	0.41 ± 0.04	330 ± 2	84 ± 6	0.16 ± 0.01	378 ± 1	20 ± 1
	0.22	6	0.44 ± 0.03	322 ± 2	80 ± 5	0.16 ± 0.01	366 ± 1	20 ± 1
	0.33	7.1	0.43 ± 0.03	312 ± 2	76 ± 5	0.19 ± 0.01	356 ± 1	22 ± 1
	0.43	8.3	0.42 ± 0.03	305 ± 2	70 ± 5	0.20 ± 0.02	347 ± 1	22 ± 1
	0.54	9.7	0.44 ± 0.03	295 ± 2	70 ± 4	0.22 ± 0.02	337 ± 1	22 ± 1
	0.75	15.1	0.54 ± 0.03	270 ± 1	69 ± 3	0.18 ± 0.01	307 ± 1	22 ± 1
0.2	0.11	3.3	0.51 ± 0.03	327 ± 1	65 ± 3	0.12 ± 0.02	365 ± 1	23 ± 2
	0.22	4.6	0.50 ± 0.03	313 ± 1	59 ± 3	0.17 ± 0.02	350 ± 1	24 ± 2
	0.33	5.7	0.50 ± 0.02	303 ± 1	54 ± 2	0.19 ± 0.02	341 ± 1	22 ± 1
	0.43	7.4	0.49 ± 0.01	291 ± 1	48 ± 1	0.19 ± 0.01	330 ± 1	21 ± 1
	0.54	9.2	0.51 ± 0.01	284 ± 1	47 ± 1	0.18 ± 0.01	323 ± 1	22 ± 1
	0.75	17	0.53 ± 0.01	245 ± 1	37 ± 1	0.21 ± 0.01	281 ± 1	26 ± 1
						(0.04 ± 0.01	323 ± 1	17 ± 3)^a^
0.4	0.11	2.4	0.52 ± 0.01	320 ± 1	28 ± 1	0.11 ± 0.01	348 ± 1	16 ± 1
	0.22	3.9	0.55 ± 0.01	304 ± 1	25 ± 1	0.13 ± 0.01	336 ± 1	20 ± 1
	0.33	5.5	0.55 ± 0.01	290 ± 1	22 ± 1	0.16 ± 0.01	324 ± 1	30 ± 2
	0.43	7.9	0.81 ± 0.02	278 ± 1	17 ± 1	0.03 ± 0.01	336 ± 1	25 ± 3
	0.54	10.5	0.70 ± 0.02	262 ± 1	15 ± 1	0.06 ± 0.01	332 ± 1	23 ± 3
	0.75	19.6	0.85 ± 0.02	232 ± 1	15 ± 1	0.09 ± 0.01	323 ± 1	22 ± 2
0.55	0.11	2.3	0.58 ± 0.01	302 ± 1	16 ± 1	0.13 ± 0.01	342 ± 1	23 ± 2
	0.22	4.4	0.68 ± 0.01	286 ± 1	11 ± 1	0.13 ± 0.01	341 ± 1	19 ± 1
	0.33	6.4	0.72 ± 0.02	269 ± 1	11 ± 1	0.10 ± 0.01	335 ± 1	18 ± 1
	0.43	9.2	0.75 ± 0.02	259 ± 1	11 ± 1	0.12 ± 0.01	333 ± 1	21 ± 1
	0.54	12	0.81 ± 0.02	247 ± 1	11 ± 1	0.12 ± 0.01	328 ± 1	20 ± 1
	0.75	22.9	0.68 ± 0.03	216 ± 1	9 ± 1	0.09 ± 0.01	315 ± 1	21 ± 2
0.75	0.11	1.9	0.72 ± 0.02	301 ± 1	9 ± 1	0.09 ± 0.01	350 ± 1	19 ± 2
	0.22	4.2	0.72 ± 0.02	286 ± 1	8 ± 1	0.09 ± 0.01	345 ± 1	19 ± 2
	0.33	6.2	0.72 ± 0.03	273 ± 1	7 ± 1	0.09 ± 0.01	340 ± 1	19 ± 1
	0.43	7.6	0.85 ± 0.02	258 ± 1	9 ± 1	0.12 ± 0.01	334 ± 1	23 ± 1
	0.54	11.8	0.61 ± 0.07	246 ± 1	8 ± 1	0.09 ± 0.01	331 ± 1	20 ± 2
	0.75	24.2	0.80 ± 0.04	214 ± 1	7 ± 1	0.18 ± 0.01	325 ± 1	25 ± 2

a Parameters of a third resolved glass transition in the Q′_S_ = 0.2, aw = 0.75 HMS-S blend.

**Table 4:** Water activity and parameters associated with the glass transition fitting, as described in Section 2.4 of [2], for the oven-dried HMS-S blends. Q׳s is the weight fraction of sucrose in the HMS-S blends (on anhydrous basis), *a*_w_ is the water activity of the matrices, ∆*C*_p,lower_ and ∆*C*_p,upper_ are the changes in heat capacity associated with the lower and upper glass transitions, *T*_g,lower_ and *T*_g,upper_ are the glass transition temperatures and of the sucrose-rich and the HMS-rich phases, respectively, and ∆*T*_g,lower_ and ∆*T*_g,lower_ are the widths of the two glass transitions.

***Q*׳**_**S**_**[dimensionless]**	***a***_**w**_**[dimensionless]**	**∆*C***_**p,lower**_**[J g**^**−1**^**K**^**−1**^**]**	***T***_**g,lower**_**[K]**	**∆*T***_**g,lower**_**[K]**	**∆*C***_**p,upper**_**[J g**^**−1**^**K**^**−1**^**]**	***T***_**g,upper**_**[K]**	**∆*T***_**g,upper**_**[K]**
0	0.014	–	–	–	0.16 ± 0.01	449 ± 4	15 ± 1
0.1	0.01	0.31 ± 0.05	390 ± 7	140 ± 20	0.08 ± 0.01	430 ± 1	15 ± 1
0.2	0.013	0.40 ± 0.02	361 ± 1	74 ± 3	0.09 ± 0.01	396 ± 1	16 ± 1
0.4	0.031	0.46 ± 0.01	331 ± 1	33 ± 1	0.08 ± 0.01	357 ± 1	19 ± 1
0.55	0.112	0.53 ± 0.01	310 ± 1	20 ± 1	0.11 ± 0.01	342 ± 8	27 ± 3
0.75	0.163	0.60 ± 0.01	301 ± 1	13 ± 1	0.07 ± 0.01	350 ± 1	20 ± 2

**Fig. 2:**
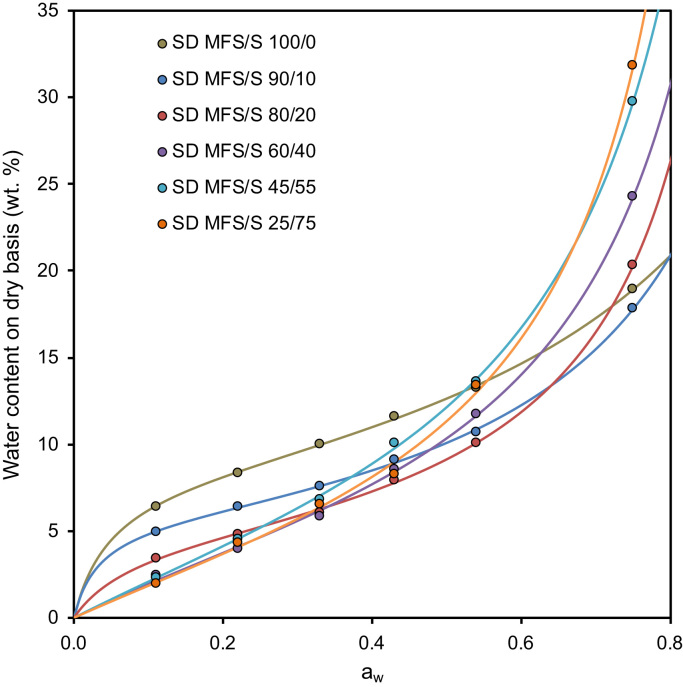
Water vapor sorption isotherms of the HMS-S blends at *T* = 298 K fitted with the GAB equation. The fitting coefficients are collected in Table 2.

The water vapor sorption data in Fig. 2 are fitted by the GAB equation (Fig. 2):

$$Q_{w}^{\prime }=\frac{KCW_{m}a_{w}}{\left(1-Ka_{w})\cdot (1-Ka_{w}+KCa_{w}\right)}$$

where *K*, *C* and *W*_m_ are fitting coefficients [3].

## 2 Experimental design, materials, and method

HMS-S blends were prepared by spray drying aqueous dispersions with well-defined ratios of HMS and S [2]. The blends were then equilibrated at a range of water activities (a_w_) at *T* = 298 K in desiccators containing saturated salt solutions (a_w_ (salt) = 0.11 (LiCl), 0.22 (CH_3_COOK), 0.33 (MgCl_2_), 0.43 (K_2_CO_3_), 0.54 (Mg(NO_3_)_2_), 0.75 (NaCl). The pure spray-dried HMS (Q′S = 0.0) was also equilibrated at *a*_w_ = 0.68 (KI)). The water activities are given by Greenspan [1]. Water sorption was followed gravimetrically for 1200 h. In this time, all samples reached their equilibrium water content. The water content of the blends was determined from the weight loss/gain upon water activity equilibration and the initial water content of the blends. These initial water contents were Initial water contents of the HMS-S blends were determined by dehydration in a laboratory oven for 27 h at 253 K at a pressure below 25 mbar and under a slight flow of dry nitrogen. Powder diffraction patterns were collected using a Phillips X׳pert Pro diffractometer (Panalytical) operating at 40 kV and 30 mA utilizing Cu Kα radiation (*λ* = 0.154 nm). Scans were performed at 298 K under local atmospheric humidity over the 2*θ* range 5–35° with a step size of 0.02° and a data acquisition time of 2 s at each step. Glass transition temperatures were determined from the 2nd heating ramp of experiments carried out by Differential Scanning Calorimetry (DSC) as described by [2]. The midpoint glass transitions were extracted from the thermograms by deconvolution assuming the presence of multiple glass transitions each characterized by a Gaussian line shape of the first derivative of the heat flow curve [2].
