# Supplementary material for: Water vapor sorption and glass transition temperatures of phase-separated amorphous blends of hydrophobically-modified starch and sucrose
Source: Data Brief. 2018 Sep 11;20:1884–9. doi: 10.1016/j.dib.2018.08.105 (PMC6172412; doi:10.1016/j.dib.2018.08.105)
Supplement: Supplementary file 1 — Supplementary material. [file mmc1.docx]

**Conflict of Interest Statement**

Manuscript number: DIB-D-18-01510

Corresponding author: J. Ubbink

Declaration of interest: Partial funding was received from DSM Nutritional Products Ltd. to enable the project that has led to the current published data.
